# Chest physiotherapy in children with acute bacterial pneumonia

**DOI:** 10.4102/sajp.v71i1.256

**Published:** 2015-09-28

**Authors:** Lieselotte Corten, Jennifer Jelsma, Brenda M. Morrow

**Affiliations:** 1 Department of Health and Rehabilitation Sciences, University of Cape Town, South Africa; 2 Department of Paediatrics and Child Health, University of Cape Town, South Africa

## Abstract

**Background:**

Pneumonia is the single leading cause of death in children younger than 5 years of age. Chest physiotherapy is often prescribed as an additional therapy in children with pneumonia. Different chest physiotherapy techniques are available that aim to improve airway clearance, gas exchange and reduce the work of breathing. However, it is unclear if these techniques are effective in this population.

**Objective:**

The present review aimed to determine the efficacy of different chest physiotherapy techniques compared with no physiotherapy or other chest physiotherapy treatments in hospitalised children with bacterial pneumonia.

**Method:**

Six electronic databases (PubMed, Medline, Cochrane Library, PEDro, CINAHL and Africa-wide information), clinicaltrials.gov and pactr.org were searched for eligible studies.

**Results:**

Two randomised controlled trials and one ongoing study were identified. Neither completed trial reported differences between the control and intervention groups, although one study reported a longer duration of coughing (*p* = 0.04) and rhonchi (*p* = 0.03) in the intervention group.

**Conclusion:**

Because of the limited number of included articles and different presentations of outcome measures, we could not reject or accept chest physiotherapy as either an effective or harmful treatment option in this population.

## Introduction

### Description of the condition

Pneumonia is an acute respiratory infection, characterised by painful breathing and limited oxygen intake as a result of fluid and pus in the alveoli (World Health Organization [WHO] 2012; WHO/UNICEF 2009). In children less than 5 years of age, pneumonia is the single leading cause of mortality, with a mortality rate of 18% globally (Mathers, Boerma & Ma Fat 2008; WHO 2012). Treatment of pneumonia consists of interventions in 3 domains: (1) protection for example, breastfeeding during the first 6 months of life, (2) prevention by vaccination and (3) appropriate antibiotic and/or symptomatic treatment (WHO 2012; WHO/UNICEF 2006, 2009). The risk of long-term respiratory sequelae after childhood pneumonia is 5.5%, with restrictive lung diseases being the most common (Edmond *et al.* 2012). However, children infected with adenovirus pneumonia can suffer from chronic obstructive lung disease as a consequence of the acute infection (Edmond *et al.* 2012). Bronchiectasis has been reported in children following hospitalisation with pneumonia (Edmond *et al.* 2012). This group of patients are reported to have three times greater risk of more severe sequelae of pneumonia (Edmond *et al.* 2012).

### Description of the intervention

In respiratory diseases such as pneumonia, increased volume and viscosity of pulmonary secretions, ciliary dyskinesia and ineffective cough may lead to reduced clearance of pulmonary secretions (Fink 2007); this predisposes to airway obstruction, inhomogeneity of ventilation, superadded infection and ultimately chronic disease such as bronchiectasis (Hardy 1994). Bacterial pneumonia may lead to mucociliary dysfunction by influencing the ciliary beat frequency (Salathé, O’Riordan & Wanner 1997); this may be a result of leukocyte production owing to host defence responses and/or bacterial products that directly or indirectly influence the ciliary beat frequency (Salathé *et al.* 1997). Standard care for patients with pneumonia is antibiotic treatment and symptomatic therapy, including oxygen support, fluid therapy, and chest physiotherapy and/or suctioning to evacuate mucus from the airways, improve ventilation and reduce the work of breathing (Principi & Esposito 2011; Wallis & Prasad 1999).

There are different types of chest physiotherapy modalities. Conventional chest physiotherapy techniques consist of positioning or postural drainage (PD), which uses gravity to eliminate mucus from the lungs (Blake 1983; Wong *et al.* 1977) and can be combined with percussions and/or vibrations on the thoracic wall to loosen secretions in the lungs; chest wall shaking; huffing and coughing (Hardy 1994; Yang, Yuping & Yin 2010). However, there is no evidence for the use of these techniques to evacuate mucus from the peripheral lung regions (Eid *et al.* 1991; Van der Schans, Piers & Postma 1986; Wong *et al.* 1977) and some serious adverse events have been reported (Button *et al.* 1997; Campbell, O’Connell & Wilson 1975; Chalumeau *et al.* 2002; Giles *et al.* 1995; Gosselink & Decramer 2001; Naylor *et al.* 2005; Selsby 1989). Newer airway clearance techniques, such as the forced expiratory technique (FET), active cycle of breathing technique (ACBT), positive expiratory pressure (PEP) technique (with or without oscillation) and autogenic drainage (AD), were developed in response to these adverse events and may promote clearance from the lungs in different ways. FET uses one or two huffs followed by a period of relaxation and controlled diaphragmatic breathing (Hardy 1994; McIlwaine 2007). The ACBT uses cycles of breathing control, thoracic expansion exercises and FET to remove secretions from the airways (Hardy 1994; Pryor 1999). During PEP therapy, positive pressure is created in the airways by breathing out against a resistance (Hardy 1994); this theoretically allows air to accumulate distally to obstructive secretions, via collateral ventilation channels (Hardy 1994). The application of AD requires patients to breathe at different lung volumes to create optimal airflow in multiple airway generations of the lung, in order to enhance secretion mobilisation from the peripheral to central airways (Chevailler 1984).

### Significance of this review

Pneumonia is the most important respiratory disease in developing countries, with an incidence of approximately 0.29 episodes per child-year in children less than 5 years of age (Rudan *et al.* 2008). Chest physiotherapy may be an appropriate tool to help airway clearance in these patients and is therefore often prescribed in patients with pneumonia. A systematic review on chest physiotherapy in adults with pneumonia (Yang *et al.* 2010) concluded that in this population, chest physiotherapy should not be given in addition to standard treatment as there is limited evidence that the techniques investigated in the review (conventional chest physiotherapy, PEP, ACBT and osteopathic manipulative techniques) have positive effects on mortality rate, duration of hospitalisation, cure rate and rate of chest X-ray improvement. A recent systematic review on chest physiotherapy in children with pneumonia (Chaves *et al.* 2013) also concluded that there was insufficient evidence to make a clear conclusion supporting or refuting chest physiotherapy in paediatric pneumonia; however, Chaves **et al.** included an article on continuous positive airway pressure which we would not classify as being a chest physiotherapy technique. Further, a non-randomised study in the paediatric population (Santos *et al.* 2009) suggested benefit from the use of chest physiotherapy, using the ’expiratory flow increase technique’, in 123 children with pneumonia. The latter study showed a significant improvement in peripheral oxygen saturation immediately after treatment, which was maintained after 20 minutes of rest (Santos *et al.* 2009). The present review therefore aimed to investigate the effects of different chest physiotherapy techniques, compared with no physiotherapy or sham physiotherapy, in hospitalised children with acute bacterial pneumonia.

## Methods

This review used the Cochrane methodology for systematic reviews (Higgins & Green 2009).

### Criteria for considering studies for the present review

We included randomised and quasi-randomised controlled trials on children under the age of 16 hospitalised with acute bacterial pneumonia. Any chest physiotherapeutic technique, as a single technique or in combination with others, was compared with no physiotherapy, sham physiotherapy or alternative therapy.

Articles were included if they were written in English, Dutch, French, German or Afrikaans. Other languages were excluded. There was no date limitation, and cross-over trials were excluded. The primary outcome measures of the present review were duration of hospital stay (days), and oxygen saturation measured before and after intervention. Secondary outcome measures were respiratory rate measured before and after intervention; duration of oxygen supplementation; lung function tests (vital capacity, forced vital capacity, forced expiratory volume in one second, peak expiratory flow, maximal inspiratory pressure and maximal expiratory pressure); any adverse effects; and mortality.

### Search methods for identification of studies

Online database searches of PubMed, Medline, Cochrane Library, PEDro, Africa-wide information and CINAHL were conducted using the following terms: (chest physiotherapy *or* chest physical therapy *or* airway clearance technique* *or* airway clearance therapy *or* breathing therapy *or* respiratory physical therapy *or* respiratory physiotherapy) *and* (child *or* children *or* infant* *or* baby *or* babies *or* toddler* *or* paediatric *or* paediatric) *and* (pneumonia *or* lung infection *or* lower respiratory tract infection *or* chest infection *or* pulmonary infection). These search terms were also translated into the different included languages by the authors.

Reference lists of the identified articles were manually checked by one of the authors (L.C.). Ongoing research was identified by exploring the clinicaltrial.gov registry and Pan African Clinical Trials registry (pactr.org). We did not search grey literature because it is very difficult to undertake a proper systematic search of the grey literature (Mahood, Van Eerd & Irvin 2014). Therefore, reproducibility of this search is challenging.

### Data collection and analysis

#### Selection of studies and data extraction

One reviewer (L.C.) searched the databases and collected relevant articles based on title and abstract; these were reviewed independently by a second reviewer (B.M.) to identify articles for full text review. Full text articles were also reviewed independently by both researchers for inclusion eligibility. Any disagreement was resolved by discussion and consensus.

Data extraction was done by two independent reviewers (L.C. and B.M.) using a pre-structured data extraction form, which included information on the participants (age, gender, condition, severity of symptoms, inclusion/exclusion criteria, comorbid conditions, setting, number randomised, number lost to follow-up); interventions (type of intervention, duration, frequency, intensity, compliance); outcome measurements; results (point estimates, precision, measures of variability, frequency counts for dichotomous variables, number of participants in each group) and study design (randomisation, allocation concealment, blinding). The data extraction form used in the present review was set up by using Chapter 8 of the Cochrane handbook: Assessing risk of bias (Higgins, Altman & Sterne 2011), the evaluation form for randomised controlled trials, and the evaluation form for systematic reviews of randomised controlled trials as found on http://dcc.cochrane.org/beoordelingsformulieren-en-andere-downloads.

#### Assessing risk of bias in included studies

One reviewer (L.C.) assessed the following methodological characteristics, which were confirmed by a second reviewer (B.M.).

**Generation of sequence:** This was considered as having low risk of bias if a random number table, computer-generated list of random numbers or any other valid method of randomisation was used. Studies were considered to have a high risk of bias when invalid methods of sequence generation were used, such as date of birth or allocation by the physiotherapist or physician. When allocation sequence generation method was not identified, bias was judged as being unclear.

**Allocation concealment:** Low risk was considered when investigators were blinded to group allocation, by the use of coded, opaque and sealed envelopes, on-site locked computer files or similar valid means. When the investigator was able to predict allocation, for example by the use of date of birth, the study was classified as having high risk of bias. When concealment details were not identified, risk of bias was considered unclear.

**Blinding:** It is generally impossible to blind the participant or clinician to most physiotherapy treatment modalities, but the physician and the data analyser could be blinded. Therefore we judged studies as having low risk of selection bias if the investigator and data analyst were blinded to treatment method. High risk of bias was considered when no blinding or a limited form of blinding was applied. Unclear risk was considered when no information on blinding was available.

**Incomplete data outcome and intention-to-treat analysis:** Low risk of bias was considered when an appropriate intention-to-treat analysis was performed on incomplete data. When no intention-to-treat analysis was conducted, data were considered as having a high risk of bias. Risk of bias was considered unclear if no information about intention-to-treat was given.

**Selective outcome reporting:** When primary and secondary outcome measures were reported, the study was considered to have low risk of bias. When no pre-specified outcome measures were identified, the risk of bias was considered high. If insufficient information was available to consider the study at high or low risk of bias, it was classified as having an unclear risk of bias.

**Other potential threats to validity:** Studies free from other threats, such as baseline imbalance or design-specific risk of bias, were considered to have low risk of bias. High risk of bias was deemed if a potential threat to validity was identified. Unclear risk of bias was deemed when insufficient information was available to determine risk of bias.

#### Measures of treatment effect

It was intended that continuous outcomes would be reported using the mean difference (or standardised mean differences) and a 95% confidence interval (CI). Where insufficient data were provided, or nonparametric measures were reported, the authors were contacted to try to obtain means (95% CI). Where this was not possible, data were reported as in the source article. Risk ratio and a 95% CI were used to report dichotomous outcomes, where possible.

## Results

### Results of the search

**FIGURE 1:**
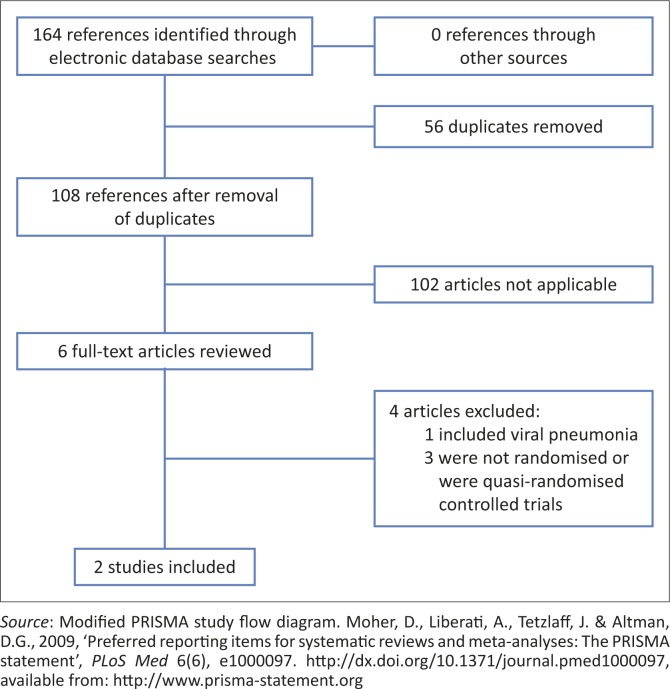
Study flow diagram.

A description of the included studies is presented in Table 1. Electronic database searches (July 2014) identified 164 articles with duplicates (45 in PubMed, 46 in Medline, 25 in PEDro, 28 in the Cochrane Library, 14 in CINAHL and 6 in Africa-wide information) (Figure 1). After removal of duplicates, 108 articles remained for further investigation. After inspection of titles and abstracts, 6 titles were considered potentially relevant and were selected for full text review. However, only 2 articles met the inclusion criteria (Lukrafka *et al.* 2012; Paludo *et al.* 2008). One ongoing randomised clinical trial was identified on pactr.org and could be included in future reviews (Appendix 1).

**TABLE 1:** Characteristics of included studies.

Characteristics	Specific characteristics	Paludo **et al.** (2008)	Lukrafka **et al.** (2012)
Methods	Study design	Randomised controlled trial	Randomised controlled trial
	Withdrawal/drop-outs	9	7
Participants	Country	Brazil	Brazil
	Research setting	Hospital	Hospital
	Health condition	Acute pneumonia	Acute CAP
	Severity of symptoms	Mild to moderate	Mild to moderate
	Total sample enrolled	98	79
	Total sample analysed	89	72
	Age range	29 days – 12 years	1–12 years
	Inclusion criteria	Acute pneumonia with:presence of cough and/or fever; tachypnoea; consolidations and/or infiltrates on CXR between 29 days and 12 years old	Hospitalised with acute CAP (clinically and radiologically diagnosed), age 1–12 years
	Exclusion criteria	Chest drain; haemodynamic instability (ND); bone fragility or rib fractures; any other contra-indication to chest physiotherapy (ND)	Severely ill patients (ICU); chest drain; atelectasis detected by CXR; history of pneumonia or pleural effusion in previous 6 months; other pulmonary disease; heart disease; CP or immune deficiency
Interventions: Intervention group	Treatment description	Standard treatment and chest physiotherapy: PD, thoracic squeezing, percussions, vibrations, cough stimulation + aspiration/suctioning when necessary. PD positions guided by CXR	< 5 years: positioned in high side lying or high sitting, manual thoracic vibrations, thoracic compressions, PEP + artificially stimulated cough or suctioning
> 5 years: same as above + breathing exercises and FET			
	Duration of treatment	About 30 minutes per treatment session	10–12 minutes per treatment session
	Frequency of treatment	Twice a day until discharge	Three times a day until discharge
	Intensity of treatment	Unclear	Unclear
	Compliance to treatment	Unclear	Unclear
Interventions: Control group	Treatment description	Standard treatment: antibiotics, fluid therapy and oxygen therapy as needed	Recommended non-mandatory request: lateral positioning, cough, perform diaphragmatic breathing
	Duration of treatment	Information not available	5 minutes (not mandatory)
	Frequency of treatment	Information not available	Once a day (not mandatory)
	Intensity of treatment	Unclear	Unclear
	Compliance to treatment	Unclear	Unclear
Outcomes	Primary outcomes	Time to clinical resolution	Severity score and respiratory rate
	Secondary outcomes	Length of hospital stay, persistence of respiratory symptoms and signs	Duration of hospitalisation

Note: Please see the full reference list of the article, Corten, L., Jelsma, J. & Morrow, B.M., 2015, ‘Chest physiotherapy in children with acute bacterial pneumonia’, *South African Journal of Physiotherapy* 71(1), Art. #256, 10 pages. http://dx.doi.org/10.4102/sajp.v71i1.256, for more information.

CAP, community-acquired pneumonia; CXR, chest X-ray; ND, not defined; ICU, intensive care unit; CP, cerebral palsy; PD, postural drainage; PEP, positive expiratory pressure; FET, forced expiratory technique.

### Included studies

Both studies included in the present review were randomised controlled trials conducted in a hospital setting in Brazil (Lukrafka *et al.* 2012; Paludo *et al.* 2008). Both articles were written in English (Lukrafka *et al.* 2012; Paludo *et al.* 2008).

#### Participants

In total, 177 participants between the age of 29 days and 12 years were enrolled in the two trials. Sixteen were lost to follow-up, and therefore 161 participants were analysed (95 male and 66 female), with 82 participants in the intervention groups and 79 in the control groups. One study (Lukrafka *et al.* 2012) divided the participants into two age groups: children younger than 5 years of age and children older than 5 years. The latter study included participants with acute community-acquired pneumonia (Lukrafka *et al.* 2012) whilst the other study did not specify acquisition site (Paludo *et al.* 2008). Both studies included participants with mild to moderate disease, but only one study clearly indicated disease severity (Lukrafka *et al.* 2012). In the other study, disease severity can be deduced as mild to moderate from the baseline characteristics of participants, as mean oxygen saturation was above 95% and mean respiratory rate at baseline was above 45 breaths per minute (Paludo *et al.* 2008) (Table 2).

**TABLE 2:** Baseline characteristics of included studies.

Characteristics	Paludo **et al.** (2008)		Lukrafka **et al.** (2012)	
	**Intervention**	**Control**	**Intervention**	**Control**
Analysed (n)	47	42	35	37
Male (n)	29	24	20	22
Female (n)	18	18	15	15
Age (n)	Mean = 44 months (95% CI 31.6–56.4)	Mean = 32.2 months (95% CI 22.5–41.9)	12–59 months: 25 (71.4%)	12–59 months: 28 (75.7%)
	-	-	5–12 years: 10 (28.6%)	5–12 years: 9 (24.3%)
Respiratory rate: mean ± s.d. (95% CI)	45 BPM ± 14.33 (40.9–49.1)	45.8 BPM ± 14.19 (41.6–50.1)	39.1 BPM ± 9.9 (35.82–42.38)	38.3 BPM ± 9.9 (35.11–41.49)
Fever (n) (%)	45 (95.7%)	37 (90.2%)	7 (20.0%)	8 (21.6%)
SaO2: mean ± s.d. (95% CI)	95.0 ± 2.47 (94.3–95.7)	95.7 ± 2.33 (95.0–96.4)	96.5 ± 2.5 (95.67–97.33)	97.1 ± 2.1 (96.42–97.78)
Pleural effusion (n) (%)	5/45 (11.1%)	6/39 (15.4%)	10 (28.6%)	4 (10.8%)

Note: Please see the full reference list of the article, Corten, L., Jelsma, J. & Morrow, B.M., 2015, ‘Chest physiotherapy in children with acute bacterial pneumonia’, *South African Journal of Physiotherapy* 71(1), Art. #256, 10 pages. http://dx.doi.org/10.4102/sajp.v71i1.256, for more information.

^95^% CI, 95% confidence interval; s.d., standard deviation; BPM, breaths per minute; SaO2, oxygen saturation.

#### Intervention

One trial (Paludo *et al.* 2008) compared standard treatment, consisting of antibiotic treatment, fluid therapy and oxygen therapy when needed, with standard treatment combined with chest physiotherapy, which included chest X-ray guided PD positioning, thoracic squeezing, percussions, vibrations, cough stimulation and aspiration/suctioning when necessary. Chest physiotherapy was given bi-daily for an average of 30 minutes per session. The other trial (Lukrafka *et al.* 2012) compared recommended non-mandatory lateral positioning, cough and the performance of diaphragmatic breathing for 5 minutes per day in the control group, with chest physiotherapy in the intervention group. In the intervention group, treatment depended on the child’s age. Participants younger than 5 years were positioned in high side lying or high sitting positions, and manual thoracic vibrations, thoracic compressions, PEP technique and artificially stimulated cough or suctioning were performed. For participants older than 5 years, the same treatment was applied with the addition of breathing exercises and FET. Treatment was given three times a day for 10–12 minutes. It is unclear how diaphragmatic breathing was taught or administered in the younger children.

### Excluded studies

Four articles did not meet the inclusion criteria for the present review. One article was excluded because participants included 55 children with presumed viral pneumonia (Levine 1978). The other three articles were excluded because the type of research was not a randomised or quasi-randomised controlled trial (Gilchrist 2008; Lisy 2014; Stapleton 1985). Stapleton (1985) described a case series of 55 children in which 34 children with acute uncomplicated respiratory tract infections received chest physiotherapy whereas 21 children with this disease did not receive chest physiotherapy and of whom 26 were diagnosed with pneumonia, 9 with bronchitis and 20 with bronchiolitis. Gilchrist (2008), on the other hand, performed a database search of the Cochrane Library, PubMed and PEDro for an answer to the structured clinical question, ‘In a child with community-acquired pneumonia, does chest physiotherapy reduce the length of hospital admission?’ Finally, Lisy (2014) presented a summary of the review by Chaves *et al.* (2013).

### Risk of bias in included studies

**FIGURE 2:**
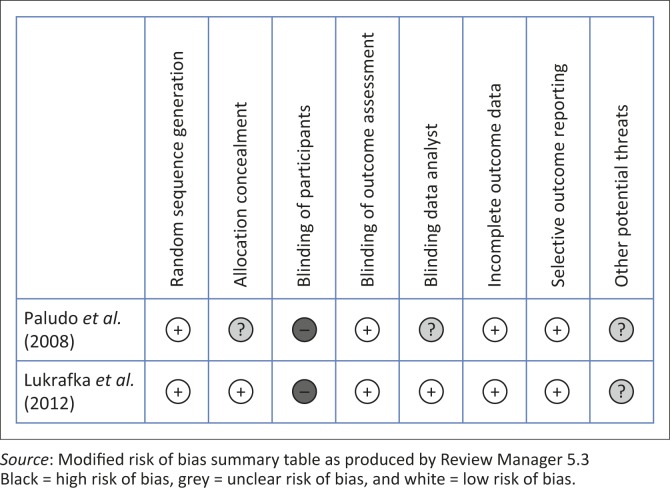
Risk of bias summary: review of authors’ judgements about each risk of bias item for each included study.

A detailed risk of bias analysis is presented in Table 3. A summary of the findings appears in Figure 2. For both studies (Lukrafka *et al.* 2012; Paludo *et al.* 2008), low risk of bias was found with regard to generation of sequence, blinding of outcome assessors, incomplete data outcome and selective outcome reporting. As it is nearly impossible to blind participants for treatment when performing chest physiotherapy, it follows that both studies have a high inherent risk of bias. Neither study commented on other potential threats to validity. Paludo *et al.* (2008) did not discuss allocation concealment or data analyst blinding.

**TABLE 3:** Risk of bias.

Category of bias	Paludo **et al.** (2008)		Lukrafka **et al.** (2012)	
	Authors’ judgement	Support for judgement	Authors’ judgement	Support for judgement
Generation of sequence	Low risk	Simple randomisation Table of random numbers	Low risk	Computerised random number generator to select blocks of 3 and 4
Allocation concealment	Unclear risk	No specifications on concealment	Low risk	Use of sequentially numbered opaque envelopes
Blinding:				
Participants	High risk	Participants knew in which group they were assigned	High risk	Participants knew in which group they were assigned
Outcome assessor	Low risk	Investigators, nurses and physicians were blinded	Low risk	Study radiologist and epidemiologist blinded
Data analysts	Unclear risk	No information on data analysts	Low risk	Data analysts are blinded
Incomplete data	Low risk	Intention-to-treat principle applied Number lost to follow-up and reason for loss to follow-up similar for both groups	Low risk	Intention-to-treat analyses Number lost to follow-up and reason for loss to follow-up similar for both groups
Selective outcome reporting	Low risk	Primary and secondary outcome measures reported	Low risk	Primary and secondary outcome measures reported
Other potential threats	Unclear risk	Baseline characteristics similar Groups treated equally, except for treatment No other information available	Unclear risk	Baseline: tendency for more children with pleural effusion in intervention group Groups treated equally, except for treatment No other information available

Note: Please see the full reference list of the article, Corten, L., Jelsma, J. & Morrow, B.M., 2015, ‘Chest physiotherapy in children with acute bacterial pneumonia’, *South African Journal of Physiotherapy* 71(1), Art. #256, 10 pages. http://dx.doi.org/10.4102/sajp.v71i1.256, for more information.

### Effects of intervention

No analysis of heterogeneity, meta-analysis or pooling of data was possible owing to different outcome measures presented in the two included studies. Duration of hospital stay data, whilst common to both included studies, could also not be pooled owing to different data presentation.

#### Primary outcomes

Both included studies reported length of hospital stay as a secondary outcome measure. In both studies, median number of days in hospital was reported and no significant difference between the groups was found (*p* = 0.76 and *p* = 0.11). In one study (Paludo *et al.* 2008), the reported median hospital stay was 6 days for both groups but, after we consulted the authors, the following additional information was made available: mean duration of stay for the intervention group was 7.8 days with a 95% CI of 6.6–9.0 days and a mean of 6.8 days for the control group with a 95% CI of 5.9–7.7 days. The other trial (Lukrafka *et al.* 2012) reported a median of 8 days in hospital for the intervention group (95% CI 5.1–10.9 days) and 6 days for the control group (95% CI 5.1–6.9 days). We were unable to obtain mean values of duration of hospitalisation for this latter trial, therefore we were unable to pool data or perform a meta-analysis.

The other primary outcome measure of the present review (oxygen saturation measured before and after intervention) was not reported in either of the included studies.

#### Secondary outcomes

Neither study reported data on any of the present review’s secondary outcome measures, namely: respiratory rate measured before and after intervention, duration of oxygen supplementation, lung function tests, adverse effects and mortality.

#### Other outcome measures

One of the studies reported time to clinical resolution, expressed in days, as their primary outcome measure (Paludo *et al.* 2008). No significant difference was seen between the intervention and control groups (*p* = 0.8). The median time to clinical resolution was 4 days in both groups, with an interquartile range of 2.0–7.0 in the intervention group and 3.0–6.0 in the control group. After consulting the authors, mean values and 95% CIs were made available. The mean time to clinical resolution in the intervention group was 4.4 days, with a 95% CI of 3.3–5.6 and 4.3 days in the control group, with a 95% CI of 3.4–5.4 (Table 4).

**TABLE 4:** Other outcome measures.

Outcome	Study (*n*)	Outcome measure	Data presentation	Intervention	Control	*p*
Primary outcomes	Paludo *et al.* (2008), n = 89	Time to clinical resolution in days	Median (IQR)	4.0 (2.0–7.0)	4.0 (3.0–6.0)	0.8
			Mean (95% CI)	4.4 (3.3–5.6)	4.3 (3.4–5.4)	n/a
	Lukrafka *et al.* (2012), n = 72	Reduction of respiratory rate	Mean ± s.d. (95% CI) at baseline	39.1 ± 9.9 (35.82–42.38)	38.4 ± 9.8 (35.24–41.56)	0.9
			Mean ± s.d. (95% CI) at discharge	31.6 ± 6.9 (29.31–33.89)	32.5 ± 8.3 (29.83–35.17)	0.7
			p value within group	p < 0.001	p < 0.001	-
	Lukrafka *et al.* (2012), n = 72	Score of severity	Mean ± s.d. (95% CI) at baseline	2.11 ± 1.6 (1.58–2.64)	1.78 ± 1.1 (1.43–2.13)	0.2
			Mean ± s.d. (95% CI) at discharge	0.57 ± 0.8 (0.31–0.84)	0.41 ± 0.6 (0.22–0.60)	0.6
			p value within group	p < 0.001	p < 0.001	-
Secondary outcomes	Paludo *et al.* (2008), n = 89	Persistence of respiratory symptoms in days	-	-	-	-
		1. Time to normal respiratory rate	Median (IQR)	3.0 (0–7.0)	3.0 (1.0–6.0)	0.75
			Mean (95% CI)	3.6 (2.4–4.8)	3.3 (2.2–4.4)	n/a
		2. Time to normal arterial SaO2	Median (IQR)	1.0 (0–2.0)	0.5 (0–2.0)	0.98
			Mean (95% CI)	1.0 (0.5–1.4)	0.8 (0.4–1.3)	n/a
		3. Time to normal lung auscultation	Median (IQR)	4.0 (3.0–6.0)	4.0 (2.0–6.0)	0.28
			Mean (95% CI)	4.7 (3.5–5.9)	4.1 (3.1–5.0)	n/a
		4. Duration of fever	Median (IQR)	2.0 (0–2.0)	1.0 (0–3.0)	0.78
			Mean (95% CI)	1.4 (0.8–1.9)	1.5 (0.7–2.3)	n/a
		5. Duration of coughing	Median (IQR)	5.0 (4.0–8.0)	4.0 (3.0–6.0)	0.04
			Mean (95% CI)	6.1 (5.1–7.1)	4.7 (3.9–5.6)	n/a
		6. Duration of parent’s reported wheezing	Median (IQR)	1.5 (0–5.0)	1.0 (0–3.5)	0.29
			Mean (95% CI)	2.9 (2.0–3.9)	1.7 (1.0–2.4)	n/a
		7. Duration of fine crackles	Median (IQR)	0 (0–2.0)	0 (0–2.0)	0.72
			Mean (95% CI)	1.1 (0.6–1.6)	1.2 (0.5–1.8)	n/a
		8. Duration of coarse crackles	Median (IQR)	2.0 (0–4.0)	1.0 (0–3.0)	0.83
			Mean (95% CI)	2.1 (1.3–2.7)	2.0 (1.1–2.8)	n/a
		9. Duration of wheezes	Median (IQR)	0 (0–5.0)	0 (0–4.0)	0.62
			Mean (95% CI)	1.7 (1.0–2.5)	1.8 (0.8–2.7)	n/a
		10. Duration of rhonchi	Median (IQR)	2.0 (0–4.0)	0.5 (0–2.0)	0.03
			Mean (95% CI)	2.8 (1.8–3.8)	1.2 (0.5–1.9)	n/a
		11. Duration of chest indrawing	Median (IQR)	2.0 (0–3.0)	2.0 (0–3.0)	0.75
			Mean (95% CI)	1.8 (1.3–2.4)	2.0 (1.2–2.8)	n/a

Note: Please see the full reference list of the article, Corten, L., Jelsma, J. & Morrow, B.M., 2015, ‘Chest physiotherapy in children with acute bacterial pneumonia’, *South African Journal of Physiotherapy* 71(1), Art. #256, 10 pages. http://dx.doi.org/10.4102/sajp.v71i1.256, for more information.

IQR, interquartile range; 95 % CI, 95% confidence interval; s.d., standard deviation; n/a, not available.

The other study (Lukrafka *et al.* 2012) used reduction of respiratory rate and illness severity score, comparing baseline and discharge results, as primary outcome measures. Both groups showed a significant improvement in outcomes between baseline and discharge (*p* < 0.001), but there were no significant differences between the groups for these outcome measures (Table 4; at discharge, *p* = 0.7 for reduction in respiratory rate, and *p* = 0.6 for severity score).

Paludo *et al.* (2008) reported persistence of respiratory symptoms, expressed in days, as a secondary outcome measure. No significant difference between the intervention and control groups was reported (Table 4), except for a longer duration of coughing (*p* = 0.04) and a longer duration of rhonchi (*p* = 0.03) in the intervention group. The median (interquartile range) duration of coughing was 5 (4.0–8.0) days in the intervention group and 4 (3.0–6.0) days in the control group. Mean (95% CI) values for this outcome measure were 6.1 (5.1–7.1) days for the intervention group and 4.7 (3.9–5.6) days for the control group. Comparing the duration of rhonchi, the intervention group had a median (interquartile range) duration of 2 (0–4.0) days and the control group 0.5 (0–2.0) days. The authors reported a mean (95% CI) duration of 2.8 (1.8–3.8) days for the intervention group and 1.2 (0.5–1.9) days for the control group.

### Potential bias in the review process

We searched six different databases, checked the reference lists of all relevant articles and searched the clinicaltrial.gov and pactr.gov registry to identify potential studies for the present review. We contacted the authors of identified articles to obtain additional or missing data, but only those of one study (Paludo *et al.* 2008) replied. No date limitation was set and no articles were excluded owing to language, which reduces the risk of selective reporting. We might have missed studies reported in grey literature, non-peer-reviewed journals or databases, as well as studies presented at local conferences, which may lead to a potential bias. One important potential bias is the identification of the ongoing clinical trial identified through pactr.gov, as the authors of this randomised controlled trial are the same as those of the present review.

## Discussion

The present review included two randomised controlled trials of 161 participants, neither of which compared chest physiotherapy with sham physiotherapy. One study compared standard treatment for pneumonia with standard treatment with additional conventional chest physiotherapy (Paludo *et al.* 2008), whilst the other study compared recommended non-mandatory lateral positioning, cough and diaphragmatic breathing with conventional chest physiotherapy combined with PEP in all children and the FET in children more than 5 years old (Lukrafka *et al.* 2012). The latter study (Lukrafka *et al.* 2012) did not distinguish between the two age categories (younger and older than 5 years) regarding control group intervention. It is unclear whether and how diaphragmatic breathing was achieved with unco-operative young infants and children.

Chest physiotherapy was not shown to influence the duration of hospitalisation (primary outcome of the present review) on the basis of both included studies. However, Lukrafka *et al.* (2012) did report a 2-day difference between the intervention and control groups, with a longer duration of hospitalisation for the intervention group. The present study might have been underpowered to detect a significant difference between the two groups. The other outcome measures of the present review were not assessed in the included studies, therefore we cannot comment on the effectiveness of chest physiotherapy regarding these measures. Conventional chest physiotherapy was not found to have an influence on time to clinical resolution (number of days for the participant to reach afebrile state, absence of severe signs, normal respiratory rate and arterial oxygen saturation ≥ 95%) (Paludo *et al.* 2008). There was also no influence of conventional chest physiotherapy combined with PEP (and FET in children more than 5 years old) on the reduction of respiratory rate and illness severity score (Lukrafka *et al.* 2012). Paludo *et al.* (2008) reported an increased ‘persistence of respiratory symptoms’ in the group receiving conventional physiotherapy with a longer duration of coughing and rhonchi in this group compared with the control group. The clinical relevance of this ‘persistence’ is not clear.

Risk of bias was present in both studies. Only one study reported the method of allocation concealment (Lukrafka *et al.* 2012) and a lack of blinding was found in both studies. Owing to the nature of the intervention, it is impossible to blind the participants of the research, therefore neither study commented on this factor. Although the outcome assessors were blinded in both trials, only one study (Lukrafka *et al.* 2012) reported blinding of the data analyst. There was inadequate information available to eliminate other potential threats regarding study validity.

Not all of the present review’s objectives were able to be addressed, as the studies did not comment on adverse events or mortality and we were unable to compare one chest physiotherapy technique with sham physiotherapy or another chest physiotherapy modality. Both studies combined several chest physiotherapy treatment techniques in their intervention, which makes it impossible to draw conclusions regarding individual techniques. As different outcome measures were used in the studies with different presentation of results, it was not possible to pool and compare all the data. Lukrafka *et al.* (2012) used severity scores to express baseline and discharge symptoms, but no separate reporting of the individual symptoms, such as oxygen saturation and fever, were available for analysis. Paludo *et al.* (2008) reported the duration of symptoms as median and interquartile ranges in the article but made the means and 95% CIs available for inclusion in the present review. Unfortunately, owing to the lack of comparable data, no meta-analysis was possible in the present review. Further, one study described the condition as ‘acute pneumonia’ (Paludo *et al.* 2008), whilst the other study (Lukrafka *et al.* 2012) described it as ‘community-acquired pneumonia’. It is therefore unclear whether the studies described exactly the same condition. Lastly, both studies were conducted in Brazil, which limits the generalisability of the findings.

Although two non-randomised controlled studies showed positive effects of chest physiotherapy (one within a population of children with community-acquired pneumonia [Santos *et al.* 2009] and one within the population of HIV-positive children on antiretroviral therapy [Plebani *et al.* 1997]), a recently published review on chest physiotherapy for pneumonia in children (Chaves *et al.* 2013) concluded that, although some minor improvements could be found in children receiving chest physiotherapy, they were unable to pool the data and make generalisable conclusions. However, the review by Chaves *et al.* (2013) differs from our review regarding the included types of pneumonia and the definition of chest physiotherapy: they included a study of nasal continuous positive airway pressure (CPAP), which we view as a form of non-invasive ventilation and not a chest physiotherapy modality. Our review was also able to identify a new, ongoing research trial, albeit conducted by the current authors, that might have biased the search strategy. Another published review on chest physiotherapy in adults with pneumonia (Yang *et al.* 2010) included six trials and concluded that osteopathic manipulations and PEP could reduce the length of hospitalisation; PEP might reduce the duration of fever; and osteopathic manipulations could reduce duration of antibiotic treatment. However, the overall conclusion was that there was insufficient evidence to support the use of chest physiotherapy in adults with pneumonia. Our review was limited to six databases, clinicaltrial.gov, pactr.org and reference lists of the included articles. We might have missed studies presented in non-peer-reviewed journals or databases, or studies presented at local conferences, which might have introduced bias.

## Conclusion

Owing to the limited number of included articles and the inability to pool data, it is not possible to make clear, justified recommendations for clinical practice. Therefore we cannot reject or accept chest physiotherapy as a standard treatment option in children with pneumonia. More randomised controlled trials in this field of research are urgently needed. We recommend research with adequate sample sizes (which could allow sub-analysis of different severity levels of pneumonia, age groups, etc.); single, standardised chest physiotherapy techniques; clear standardised control interventions; appropriate outcome parameters; and analysis of adverse events and mortality.

## Appendix 1

**TABLE 1-A1:** Characteristics of ongoing studies.

PACTR201404000706382	Description
Public trial name or title	The use of chest physiotherapy in children hospitalised with pneumonia
Scientific name or title	The use of assisted autogenic drainage in children with acute respiratory disease in a developing country
Methods	Randomised controlled trial
Participants	98 children between the age of 1 month and 8 years hospitalised with pneumonia
Interventions	Comparison of standard nursing care with standard nursing care + assisted autogenic drainage bi-daily
Outcome measures	Primary: duration of hospitalisation Secondary: duration of fever; respiratory rate at admission, before, immediately after, 1 hr post-treatment and at discharge; lung function test at admission and discharge if the child is older than 5 years of age; oxygen saturation at admission, before, during, after, 1 hr post-treatment and at discharge; duration of oxygen supplementation; atelectasis/collapse; progression of respiratory support; and mortality rate
Research setting	Red Cross War Memorial Children’s Hospital, Cape Town, South Africa
Starting date	24 March 2014
Contact information	Lieselotte Corten and Brenda Morrow. CRTLIE001@myuct.ac.za or Brenda.morrow@uct.ac.za
